# Disruption of Glutamate Release and Uptake-Related Protein Expression After Noise-Induced Synaptopathy in the Cochlea

**DOI:** 10.3389/fcell.2021.720902

**Published:** 2021-08-04

**Authors:** Kefeng Ma, Anran Zhang, Xiaojun She, Honglian Yang, Kun Wang, Yingwen Zhu, Xiujie Gao, Bo Cui

**Affiliations:** ^1^Tianjin Institute of Environmental and Operational Medicine, Tianjin, China; ^2^Shandong Academy of Occupational Health and Occupational Medicine, Shandong First Medical University & Shandong Academy of Medical Sciences, Jinan, China

**Keywords:** noise, synaptopathy, Vglut3, GLAST, Na^+^/K^+^-ATPase α1, glutamate excitotoxicity

## Abstract

High-intensity noise can cause permanent hearing loss; however, short-duration medium-intensity noise only induces a temporary threshold shift (TTS) and damages synapses formed by inner hair cells (IHCs) and spiral ganglion nerves. Synaptopathy is generally thought to be caused by glutamate excitotoxicity. In this study, we investigated the expression levels of vesicle transporter protein 3 (Vglut3), responsible for the release of glutamate; glutamate/aspartate transporter protein (GLAST), responsible for the uptake of glutamate; and Na^+^/K^+^-ATPase α1 coupled with GLAST, in the process of synaptopathy in the cochlea. The results of the auditory brainstem response (ABR) and CtBP2 immunofluorescence revealed that synaptopathy was induced on day 30 after 100 dB SPL noise exposure in C57BL/6J mice. We found that GLAST and Na^+^/K^+^-ATPase α1 were co-localized in the cochlea, mainly in the stria vascularis, spiral ligament, and spiral ganglion cells. Furthermore, Vglut3, GLAST, and Na^+^/K^+^-ATPase α1 expression were disrupted after noise exposure. These results indicate that disruption of glutamate release and uptake-related protein expression may exacerbate the occurrence of synaptopathy.

## Introduction

High-level noise, which can cause sensorineural hearing loss accompanied by permanent threshold shift, has become a major threat to human health. Short-duration medium-intensity noise (e.g., 98, 100, 106 dB SPL, 2 h), which only causes a temporary threshold shift (TTS), is distinguished from high-level noise exposure (Furman et al., 2013; Liberman et al., 2015; Bakay et al., 2018; Fernandez et al., 2020; Wei et al., 2020). In the inner ear, the mechanical vibration of sound wave was transformed into the electric signals by cochlear hair cells (Wang et al., 2017; Liu Y. et al., 2019; Qi et al., 2019, 2020; Zhang Y. et al., 2020); while spiral ganglion neurons mainly function as the neural auditory transduction cells (Sun et al., 2016; Guo et al., 2019, 2021; Liu W. et al., 2019b; Zhao et al., 2019). Noise induced hearing loss includes damage of cochlear hair cells (Liu et al., 2016; He et al., 2017, 2021; Zhou et al., 2020; Cheng et al., 2021; Fu et al., 2021), cochlear supporting cells (Lu et al., 2017; Cheng et al., 2019; Tan et al., 2019; Zhang S. et al., 2019, 2020; Zhang Y. et al., 2020; Chen et al., 2021), spiral ganglion neurons (Guo et al., 2016, 2020, 2021; Sun et al., 2016; Liu et al., 2021) and ribbon synaptopathy (Furman et al., 2013; Shi et al., 2013; Kujawa and Liberman, 2015; Liberman et al., 2015; Bakay et al., 2018; Fernandez et al., 2020; Kohrman et al., 2020; Tserga et al., 2020a; Wei et al., 2020; Song et al., 2021). It has been reported that the wave I amplitude of the auditory brainstem response (ABR) is permanently reduced and ribbon synapses between inner hair cells (IHCs) and spiral ganglion nerves are damaged after exposure to short-duration medium intensity noise (Liberman et al., 2015; Fernandez et al., 2020; Wei et al., 2020). Glutamate excitotoxicity is known to be a major factor in the damage to ribbon synapses (Kurabi et al., 2017; Sebe et al., 2017; Hu et al., 2020), but it is unknown how glutamate-associated proteins are altered after noise exposure.

Vesicle transporter protein 3 (Vglut3), encoded by the SLC17A8 gene, is important for the development and maturation of the inner ear (Obholzer et al., 2008; Ruel et al., 2008; Kim et al., 2019). Within IHCs, Vglut3 facilitates the packaging of glutamate into vesicles which is subsequently secreted into the postsynaptic membrane during exocytosis, to transduce acoustic signals into neural signals. Disrupted expression or deletion of Vglut3 can lead to tinnitus (Zhang W. et al., 2020) and deafness (Ruel et al., 2008; Seal et al., 2008; Akil et al., 2012); previous studies have suggested that knocking out the Vglut3 allele results in decreased ribbon synapse density and the number of spiral ganglion nerves (Kim et al., 2019). Moreover, ototoxic drugs (Zhang Y. et al., 2020) and aging (Peng et al., 2013) can also affect the expression of Vglut3.

Glutamate/aspartate transporter protein (GLAST) is widely expressed in the central and peripheral nervous systems (CNS and PNS, respectively), and is mainly expressed in the pillar cells (PCs) surrounding IHCs, the main site of glutamate uptake in the inner ear (Glowatzki et al., 2006). Hakuba et al. (2000) found that cochlear glutamate levels were much higher in GLAST^–/–^ animals compared to wild-type animals, which may result from glutamate uptake dysfunction. Glutamate uptake through the GLAST is dependent on the Na^+^ concentration gradient, which is primarily maintained by Na^+^/K^+^-ATPase (Zhang et al., 2016). Na^+^/K^+^-ATPase consists of α, β, and γ subunits, which are further divided into α1, α2, and α3 subunits. CNS studies have shown that GLAST interacts with Na^+^/K^+^-ATPase, especially through the α1 subunit to co-uptake extracellular glutamate to protect neurons from excitotoxic injury (Bauer et al., 2012; Zhang et al., 2016). It is unclear whether GLAST interacts with Na^+^/K^+^-ATPase α1 in the cochlea.

A cycle consisting of Vglut3 and GLAST maintains low concentrations of glutamate in the cochlea. Excitotoxicity may occur when this cycle is disrupted. Therefore, we wanted to study how Vglut3, GLAST, and Na^+^/K^+^-ATPase α1 changed after ribbon synaptopathy. First, we investigated whether GLAST interacts with Na^+^/K^+^-ATPase α1 in the inner ear. Second, we constructed a ribbon synaptopathy model using 100 dB SPL white noise, and then measured the expression of Vglut3, GLAST, and Na^+^/K^+^-ATPase α1 at four different time points (2 h, 1 day, 7 days, and 30 days), after exposure to noise.

## Materials and Methods

### Animals and Groups

Six-week-old male C57BL/6J mice (*N* = 55) were purchased from Vital River (Beijing, China). The animals were housed in a 12 h light/dark cycle for 1 week in an animal laboratory room, where the ambient noise was maintained below 50 dB SPL, and food and water were provided *ad libitum*. Fifteen mice were used for immunoprecipitation experiments before noise exposure, and the remaining 40 mice were divided into two groups. Ten mice were included in the control group (Ctr) without noise exposure; one mouse died due to an overdose of anesthetic injection during the ABR measurement. Thirty mice were included in the noise exposure group. ABR and immunofluorescence were detected on day 1 (1 d), day 7 (7 d), and day 30 (30 d) after noise exposure. All experiments were approved by the ethics committee of the Tianjin Institute of Environmental and Operational Medicine.

### Noise Exposure

Mice were placed in a small cage woven with wire and placed under an amplifier (IBO, BA-215, China) at a distance of 10 cm from the mouse’s ears. Mice were exposed to a 100 dB SPL white noise stimulus for 2 h produced by a sound generator (SKC, GZ009, China). The sound intensity was calibrated with a sound level meter (BSWA, 308, China) at the mouse’s ear position, from different directions. The average noise level was 100 ± 1.9 dB SPL.

### ABR Detection

Auditory brainstem response was measured in an electroacoustic shielded room before noise exposure and on days 1, 7, and 30 after noise exposure. Mice were anesthetized using ketamine (100 mg/kg) and thiazide (3 mg/kg), and the recording needle was inserted into the Fz point of the head, the reference needle was placed in the mastoid of both ears, and the grounding needle was inserted into the skin of the forepaw. ABR waveforms were recorded at 4, 8, 12, and 16 kHz pure-tone (3,000 μs) stimulations. Sound intensity was decreased in 10 dB steps at high levels of stimulation and in 5 dB steps near the hearing threshold. Because wave II was easily recognized, the lowest stimulus intensity of wave II was used as the hearing threshold, and was repeated it three times to confirm the threshold intensity. A wave I amplitude of 90 dB was detected at each frequency.

### Immunofluorescence

Mice were anesthetized with ketamine (100 mg/kg) and thiazide (3 mg/kg) and the cochleae were carefully removed and fixed with 4% paraformaldehyde (PFA). Briefly, the muscle tissue was carefully removed under a body microscope (Olympus, SZX7, Japan), and a hole was drilled at the top of the cochlea. PFA was slowly injected into the cochlea from the round window, with a syringe until the top of cochlea flowed clear liquid; the cochleae were then fixed overnight at 4°C in 4% PFA. The fixed cochlea was placed in 10% ethylene diamine tetra-acetic acid at room temperature overnight. For whole-mount staining, cochlear basilar membranes were carefully isolated and rinsed three times with Phosphate Buffered Saline (PBS). For frozen sections, cochleae were rinsed three times with PBS and dehydrated overnight in 30% sucrose. Then, cochleae were cut into 20 μm sections after incubation in embedding agent for 3 days. Tissues were incubated in PBS containing 1% Triton-X 100 for 30 min at room temperature, followed by incubation with PBS containing 0.5% Triton-X 100 and 5% BSA for 1 h at room temperature. Rabbit anti-CtBP2 (1:100; Bioworld, BS2287), rabbit anti-GLAST (1:1,000, Abcam, Ab416), and mouse anti-Na^+^/K^+^-ATPase α1 (1:500, Millipore, #05-369) were applied overnight or for 2 h at room temperature. Tissues were rinsed three times for 10 min each with PBS containing 1% Triton-X 100. Tissue sections were incubated in secondary antibodies conjugated with dylight 488 (1:500, Bioworld, BS10015) or dylight 549 (1:500, Bioworld, BS10023), for 1 h at room temperature. Tissues were rinsed three times for 10 min each with PBS containing 1% Triton-X 100. The tissues were mounted on glass slides after nuclei staining.

### Confocal and Fluorescence Microscopy

For whole-mount staining, tissues were photographed with a 63× oil objective using 546 nm wave with a laser confocal microscope (Leica, SP8, Germany). Meanwhile, the field of view was 1.8 digital zoom. All photographs were taken in 0.5 μm steps with equal laser intensity and exposure time. For frozen sections, photographs were taken under a normal fluorescence microscope (Olympus BX51, Japan). Panoramic and local photographs were taken under 20× and 100× objectives, respectively, with equal fluorescence intensity and exposure time.

### Immunoprecipitation

The basilar membrane, spiral ligament, and osseous spiral lamina, which were removed from the ear unexposed to noise, were placed in IP lysate (60 μL, containing 1% inhibitor cocktail) on ice. Tissues were homogenized with a pestle and lysed on ice for 30 min. The supernatant (approximately 50 μL) was collected after centrifugation at 10,000 rpm for 15 min at 4°C. GLAST or IgG antibody (1 μL; CST, #5684, Santa Cruz, sc-2025, respectively) and supernatant were co-incubated overnight at 4°C in a shaker. An equal volume of beads (Santa Cruz, sc-2003) were added. The mixture was incubated at room temperature for 4 h, followed by centrifugation at 3,000 rpm for 5 min at 4°C. The beads were rinsed three times with IP lysis solution, and finally resuspended in 30 μl of IP lysis solution. Western Blotting was performed after adding 30 μl of loading buffer to boil.

### Western Blot

To extract total protein, cochleae were homogenized in radioimmunoprecipitation assay buffer (containing 1% enzyme inhibitor), and the bones were removed. Samples were loaded according to total protein amount, which was calculated by measuring the gray level of β-actin (1:200, Santa Cruz, sc-47778). Proteins were separated using sodium dodecyl sulfate-polyacrylamide gel electrophoresis. After electrophoresis, the proteins were transferred onto a nitrocellulose membrane and blocked with 5% degreased milk powder in PBS plus 0.1% Tween 20 (PBST). The membrane was incubated with primary antibodies at 4°C overnight and washed three times (10 min per wash) with PBST. The membranes were then incubated with the secondary antibodies for 2 h. After the membrane was washed, the protein bands were visualized by electrochemiluminescence.

### Statistical Analysis

All data are expressed as the mean ± standard error of the mean. All statistical analyses were performed using GraphPad Prism version 8. ABR threshold and amplitude data were analyzed by two-way analysis of variance (ANOVA). CtBP2 count and protein expression levels were analyzed by one-way ANOVA.

## Results

### ABR Threshold and Wave I Amplitude Detection

**FIGURE 1:**
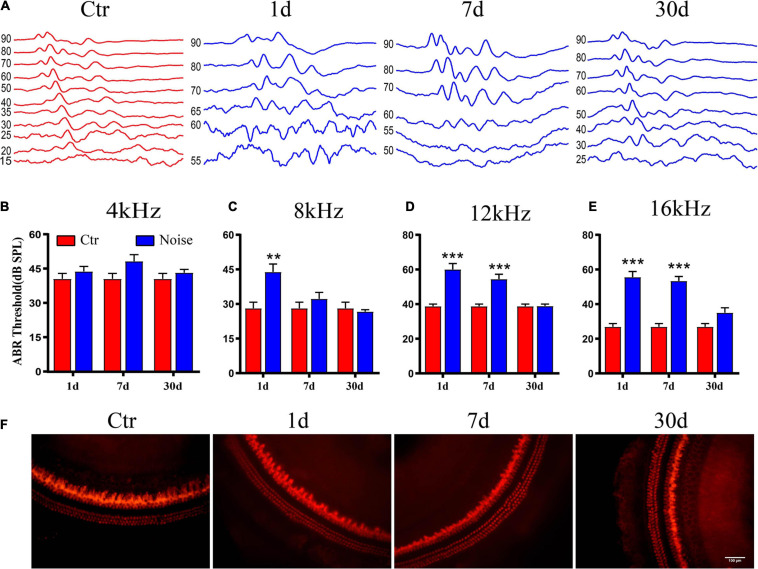
Auditory brainstem response (ABR) threshold following noise exposure. **(A)** The waveform of control and day 1, 7, and 30 after noise exposure with 90 dB SPL pure tone stimulation at 16 kHz. **(B–E)** Statistical analysis of ABR threshold showed temporary threshold shift at 4 kHz **(B)**, 8 kHz **(C)**, 12 kHz **(D)**, and 16 kHz **(E)** (*n* = 9–10). **(F)** Immunofluorescence image of hair cells at middle turn in the basilar membrane with anti-myosin VIIa antibody at 20x objective, scale bar = 100 μm. **vs. Ctr, *p* < 0.01; ***vs. Ctr, *p* < 0.001.

To examine the effects of noise on the peripheral auditory system, we first compared hearing thresholds between the control and noise groups on days 1, 7, and 30-post exposure. There was no change in the 4 kHz hearing threshold (Figure 1B). Hearing thresholds at 8 kHz were only significantly elevated on day 1 after exposure (Figure 1C), and significantly higher than that of the control on days 1 and 7 after noise exposure, at both 12 kHz and 16 kHz (Figures 1A,D,E). The thresholds of all pure tones were not significantly different from controls on day 30 post-exposure (Figures 1A–E). These ABR threshold results indicate that the TTS is caused by noise. Furthermore, hair cell damage was not detected on days 1, 7, or 30 after noise exposure, compared to the Ctr (Figure 1F).

**FIGURE 2:**
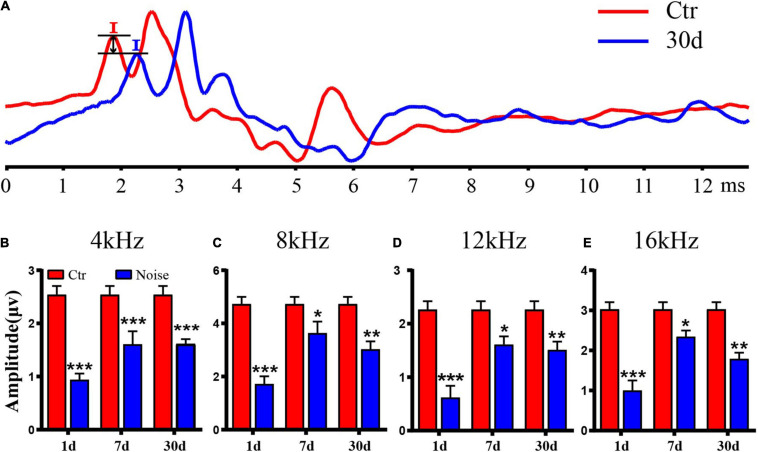
Auditory brainstem response (ABR) wave I amplitude after noise exposure. **(A)** The comparison of wave I amplitude of the control group and day 30 after noise exposure with 90 dB SPL pure tone stimulation at 16 kHz. **(B–E)** Statistical analysis of wave I amplitude at 4 kHz **(B)**, 8 kHz **(C)**, 12 kHz **(D)**, and 16 kHz **(E)** (*n* = 9–10). *vs. Ctr, *p* < 0.05; **vs. Ctr, *p* < 0.01; ***vs. Ctr, *p* < 0.001.

We then measured the change in wave I amplitude and found that the wave I amplitude of all pure tones was significantly lower than that of the Ctr (Figures 2A–E). On day 1 after noise exposure, the amplitude of wave I was the lowest at all frequencies (Figures 2B–E). On day 7, the amplitude recovered compared to day 1 (Figures 2B–E). On day 30, there was a significant decrease in amplitude compared with the Ctr, as well as a decrease compared to day 7 (Figures 2B–E). These amplitude results indicate that nerve transmission was damaged by noise exposure. It seems that the latency of wave I was delayed after noise exposure (Figure 2A). Through statistical analysis, no significant difference was found in latency of wave I on day 30 (data not show), which is consistent with previous studies (Kujawa and Liberman, 2015; Liberman et al., 2015; Fernandez et al., 2020; Kohrman et al., 2020; Wei et al., 2020; Song et al., 2021).

### Ribbon Synaptopathy Caused by Noise Exposure

**FIGURE 3:**
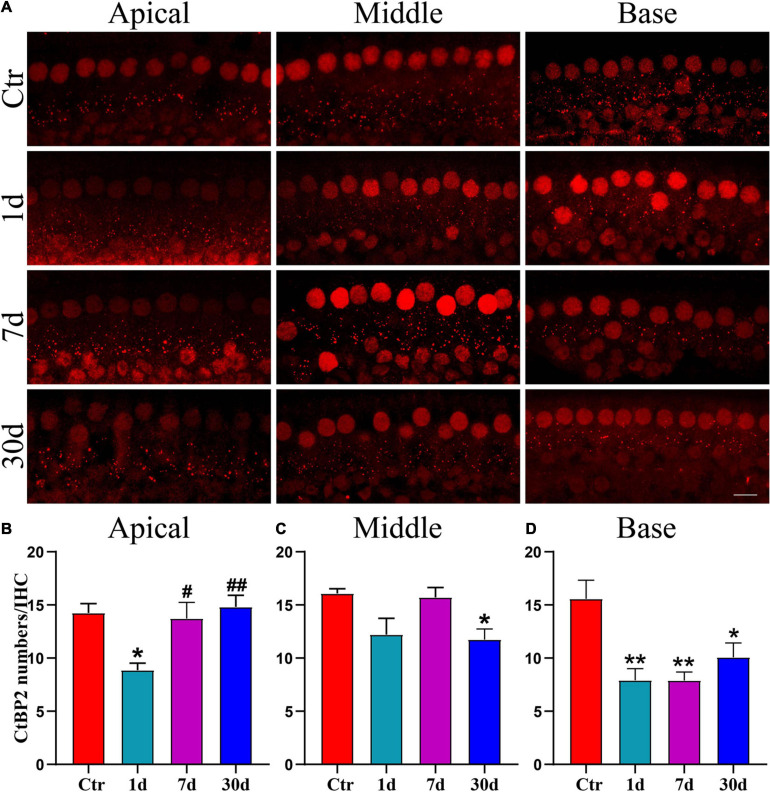
Changes of CtBP2 numbers after noise exposure. **(A)** Immunofluorescence images was taken by confocal microscope with a 63x oil objective. Punctate CtBP2 was labeled with a CtBP2 antibody in IHCs; IHCs and supporting cells’ nuclei were also stained, scale bars = 10 μm. **(B–D)** Statistical analysis of CtBP2 in each group (*n* = 4–5 ears) on day 1, day 7, and day 30 after noise exposure. *vs. Ctr, *p* < 0.05; **vs. Ctr, *p* < 0.01; #vs. 1 day, *p* < 0.05; ##vs. 1 day, *p* < 0.01.

Since the amplitude of ABR wave I indicates the total activity of the SGN (Plack et al., 2016), we stained for CtBP2 on day 1, 7, and 30 after noise exposure, to determine whether the decrease in wave I amplitude was caused by ribbon synaptopathy. Punctate CtBP2 was distributed around IHCs as well as within the nuclei, and in supporting cells (Figure 3A). In the apical region, CtBP2 numbers decreased significantly on day 1 after noise exposure; however, CtBP2 numbers recovered significantly on days 7 and 30, but this difference was not significant compared to that of day 1 (Figures 3A,B). In the middle region, the number of CtBP2 decreased on day 1 after noise exposure, but there was no significant difference compared with the Ctr. On day 7 after exposure, the numbers were equivalent to controls and recovered compared with day 1 after noise exposure. The CtBP2 numbers were significantly lower than those in the Ctr on day 30, after noise exposure (Figures 3A,C). In the apical and middle regions, the recovery of CtBP2 may suggest the presence of synaptic remodeling. In the base region, the number of CtBP2 decreased significantly on days 1, 7, and 30 after noise exposure (Figures 3A,D). The change in CtBP2 numbers is consistent with the change in wave I amplitude.

### The Interaction of GLAST and Na^+^/K^+^-ATPase α1 in Cochlea

**FIGURE 4:**
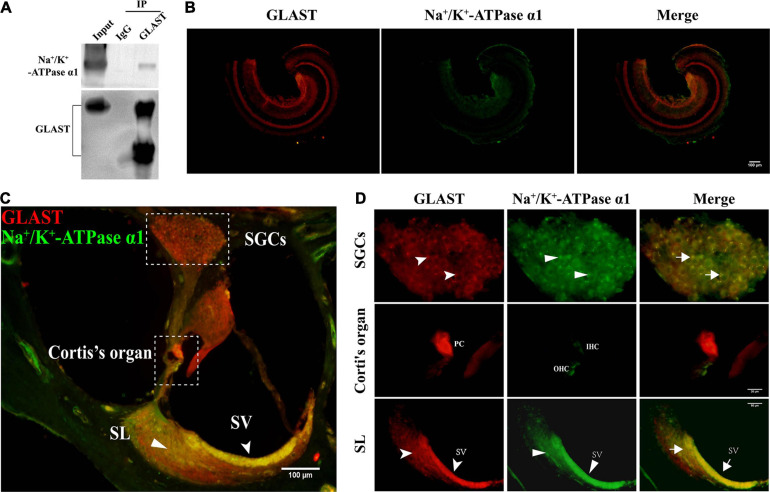
Interaction of GLAST and Na^+^/K^+^-ATPase α1. **(A)** Immunoprecipitation of GLAST from cochlear tissue. Anti-GLAST antibody or IgG was used for Immunoprecipitation from cochlea lysates (*n* = 10 ears). Except for the IgG lane, endogenous Na^+^/K^+^-ATPase α1 and GLAST were all detected on the same membrane. **(B)** Immunofluorescence image of apical turn in the basilar membrane, scale bar = 100 μm. **(C)** Frozen sections of noise-unexposed C57BL/6J cochlea were stained with anti-GLAST antibody (red) and anti- Na^+^/K^+^-ATPase α1 (green) antibody, scale bar = 100 μm; the dovetailed arrowhead represents SV; the flat-tailed arrowhead represents SL. **(D)** Corti’s organ and spiral ganglion cells (SGCs) were magnified under 100x oil objective, scale bars = 20 μm; spiral ligament is magnified under 40x objective, scale bars = 50 μm; GLAST (the dovetailed arrowhead), Na^+^/K^+^-ATPase α1 (the flat-tailed arrowhead) and merged immunopositive fluorescence (the arrow) were point out in particular. SGCs, spiral ganglion cells; SV, stria vascularis; SL, spiral ligament; PCs, pillar cells; OHCs, out hair cells; IHCs, inner hair cells.

Studies of the CNS have shown a clear interaction between GLAST and Na^+^/K^+^-ATPase α1 (Bauer et al., 2012; Zhang et al., 2016). To investigate the relationship between GLAST and Na^+^/K^+^-ATPase α1 in the cochlea, we performed immunoprecipitation and immuno co-localization within the cochlea. After purification of the protein with GLAST antibody, immunoblotting of GLAST and Na^+^/K^+^-ATPase α1 on the same membrane was performed sequentially, and clear bands were observed in the input lane and the GLAST lane, but not in the IgG lane (Figure 4A). Following, we detected immunoreactivity of GLAST and Na^+^/K^+^-ATPase α1 in the cochlear basilar membrane; only GLAST immuno-positivity was identified (Figure 4B). To determine immunoreactivity in other structures, we performed immunofluorescence using frozen sections. We found co-localization of GLAST and Na^+^/K^+^-ATPase α1 in the stria vascularis (Figure 4C, white dovetailed arrowhead, D), the spiral ligament (Figure 4C, white flat-tailed arrowhead, D), and spiral ganglion cells (Figures 4C,D). Na^+^/K^+^-ATPase α1 was only expressed in hair cells in the Organ of Cotti. There is no evidence that GLAST and Na^+^/K^+^-ATPase α1 are co-expressed in PCs which are major cells to take in glutamate (Figures 4B–D).

### Noise Exposure Disorders Vglut3, GLAST and Na^+^/K^+^-ATPase α1 Expression

**FIGURE 5:**
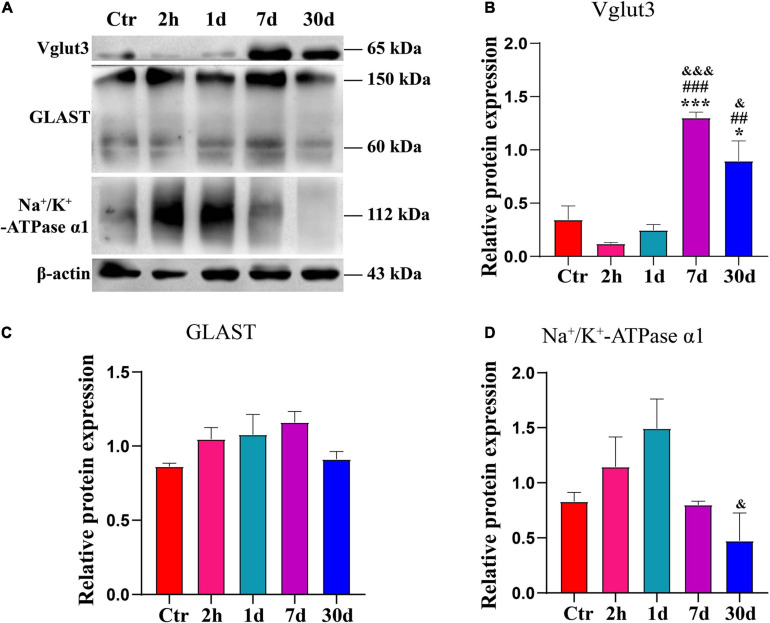
Expression levels of GLAST, Na^+^/K^+^-ATPase α1 and Vglut3 were disrupted after noise exposure. **(A)** Western blot of Vglut3, GLAST and Na^+^/K^+^-ATPase α1 from normal cochlea and noise-exposure cochlea on hour 2, day 1, day 7, day 30 after noise (*n* = 6–8 ears). **(B–D)** Quantification of expression levels of Vglut3, GLAST and Na^+^/K^+^-ATPase α1. The statistics were repeated three times. *vs. Ctr, *p* < 0.05; ***vs. Ctr, *p* < 0.001. ##vs. 2 h, *p* < 0.01; ###vs. 2 h, *p* < 0.001; &vs. 1 day, *p* < 0.05; &&&vs. 1 day, *p* < 0.001.

To determine the changes in Vglut3, GLAST or Na^+^/K^+^-ATPase α1, we examined the expression levels of these proteins at 2 h, 1 day, 7 days, and 30 days after noise exposure (Figure 5A). Vglut3 expression level was lowest at 2 h after noise exposure. Vglut3 expression level recovered on day 1, but it was still lower than that of the Ctr. On day 7, the Vglut3 expression level increased significantly compared to that of the control at hour 2 or day 1 (Figure 5B). Vglut3 expression level decreased on day 30 but was still significantly higher than that in the control at hour 2, or day 1 (Figure 5B). The expression levels of GLAST and Na^+^/K^+^-ATPase α1 were completely opposite to those of Vglut3. The expression level of GLAST gradually increased between hour 2 and day 7, it recovered until on day 30 after noise exposure (Figure 5C). The change in Na^+^/K^+^-ATPase α1 was consistent with GLAST at 2 h and 1 day after noise exposure, but returned to normal levels on day 7 after noise exposure, and significantly decreased on day 30 compared to day 1 (Figure 5D). These results indicate an enhanced ability to release glutamate and a decreased ability to uptake glutamate on day 30 after noise exposure, which may be the main mediator of synaptopathy.

## Discussion

The cochlear hair cells are sensitive to aging, acoustic trauma, ototoxic drugs, and environmental or genetic influences (Zhu et al., 2018; Fang et al., 2019; He et al., 2020; Jiang et al., 2020; Qian et al., 2020; Lv et al., 2021; Zhang et al., 2021). Previous reports have shown that oxidative stress and cell apoptosis play important roles in noise induced hair cell loss and ribbon synaptopathy (Sun et al., 2014; Yu et al., 2017; Li et al., 2018; Gao et al., 2019; He et al., 2019; Zhang Y. et al., 2019; Zhong et al., 2020). In this study, we confirmed the interaction between GLAST and Na^+^/K^+^-ATPase α1 in the cochlea of C57BL/6J mice; protein expression was mainly co-localized in the stria vascularis, spiral ligament, and spiral ganglion cells, but not in the PCs, the major site of glutamate uptake. On day 30 after noise exposure, C57BL/6J mice experienced a TTS, a decrease in wave I amplitude at the 4, 8, 12, and 16 kHz cochlear regions, and a decrease in the amount of the presynaptic protein CtBP2. Meanwhile, the expression level of Vglut3 was upregulated on day 30 after exposure, and the expression level of GLAST remained almost unchanged, however, the expression level of Na^+^/K^+^-ATPase α1, which is directly coupled to GLAST, was downregulated. These findings may reveal an intrinsic link between noise-induced glutamate excitotoxicity and ribbon synaptopathy.

Vglut3 dysfunction or deficiency disrupts nerve conduction in the peripheral auditory system. LSP5-2157, an inhibitor of Vglut3, inhibited the compound action potential of the peripheral auditory system in guinea pigs (Poirel et al., 2020). Animals lose hearing after knockout of the Vglut3 allele; however, it can be restored using an adenoviral vector delivery system to re-establish Vglut3 expression (Akil et al., 2015; Akil and Lustig, 2019; Kim et al., 2019). In our study, the expression of Vglut3 was lowest at 2 h, slightly recovered on day 1, was highest on day 7, and slightly lowered on day 30 which was almost consistent with the observed change in wave I amplitude. Hu et al. (2020) and Sebe et al. (2017) considered that noise-induced ribbon synaptopathy is caused by over-activation of Ca^2+^-permeable AMPA receptors (CP-AMPARs), which lack GluR2, and mediate excessive inward Ca^2+^ which can damage presynaptic ribbons and postsynaptic receptors. Kim et al. (2019) revealed the role of the Vglut3 in CP-AMPAR-mediated glutamatergic excitotoxicity. They found that a single copy of the Vglut3 gene was sufficient to cause ribbon synaptopathy after noise exposure, and deletion of the Vglut3 allele reduced excitotoxicity induced by noise. Our study found that the intensity of CtBP2 significantly reduced in the apical and base regions on day 1 after noise exposure, compared to that of the Ctr, and that the expression of CtBP2 in the middle region was reduced compared to the Ctr (not statistically significant). Although Vglut3 expression levels decreased on day 1 after noise exposure, the ribbon synapse structure was also damaged, which means that low levels of glutamate can also cause ribbon synaptopathy under the influence of noise. Synapses recovered on day 7 (apical and middle region) or day 30 (apical region) after exposure, suggesting the presence of synaptic reconstruction. ABR thresholds and wave I amplitudes of Vglut3^WT^ animals recovered better than Vglut3^+/−^ mice exposed to 94 dB SPL noise, indicating that Vglut3 or glutamate release contributed to hearing recovery (Kim et al., 2019). Glutamate release may also help to construct synapses (Akil et al., 2012; Shi et al., 2013; Song et al., 2021). Synaptic remodeling was not observed on day 7 in the base region, and it is possible that the base region is more susceptible to noise and the development of ribbon synaptopathy. Our results are consistent with those of the previous studies. Reduced ribbon synapses may promote the expression of Vglut3 protein to maintain physiological signal transduction.

The main function of GLAST transport extracellular glutamate, while the uptake drive depends on the Na^+^ concentration gradient inside and outside of cells; Na^+^/K^+^-ATPase maintains the Na^+^ gradient concentration difference by hydrolyzing ATP (Robinson and Jackson, 2016). Studies on astrocytes and the CNS have shown that Na^+^/K^+^-ATPase is directly coupled to GLAST *via* the α subunit (Rose et al., 2009; Bauer et al., 2012; Robinson and Jackson, 2016; Zhang et al., 2016). In the peripheral auditory system, GLAST and Na^+^/K^+^-ATPase α1 are both expressed in PCs surrounding IHCs (Glowatzki et al., 2006; McLean et al., 2009; Sundaresan et al., 2016; Liu W. et al., 2019a; Stephenson et al., 2021). In our study, we found an interaction between GLAST and Na^+^/K^+^-ATPase α1; however, immuno co-localization revealed that the sites of interaction were in the stria vascularis, spiral ligament, and SGCs. There was no α1 subunit immunoreactivity in the PCs. Immunoreactivity of Na^+^/K^+^-ATPase α1 have been demonstrated in stria vascularis, spiral ligament and support cells in the human cochlea (Stephenson et al., 2021). Our findings on Na^+^/K^+^-ATPase α1 were consistent with published researches on stria vascularis, spiral ligament, SGCs and hair cells (Clemens Grisham et al., 2013; Yamaguchi et al., 2014; Ding et al., 2018; Liu W. et al., 2019a; Stephenson et al., 2021). Our findings in PCs differ from studies done in rats (McLean et al., 2009). PCs and IHCs are adjacent in spatial structure, which maybe an account for no immunoreactivity of Na^+^/K^+^-ATPase α1 in PCs. Na^+^/K^+^-ATPase α1 not expressing simultaneously in PCs or IHCs can reduce structural and functional redundancy.

The functional deficiency of GLAST affects hearing sensitivity and synaptic integrity (Yu et al., 2016; Tserga et al., 2020b). Glutamate levels in the endolymph fluid were twice as high in GLAST knockout animals compared to wild-type mice, after noise exposure, revealing that noise-induced hearing loss and ribbon synaptopathy may be caused by glutamate excitotoxicity (Hakuba et al., 2000). The combined exogenous inhibitor and glutamate perfusion further demonstrated the importance of GLAST in the hearing system (Chen et al., 2010). In our study, GLAST expression levels gradually increased after noise exposure and recovered on day 30, possibly indicating that the inner ear prevented glutamate toxicity by increasing GLAST expression levels. However, Vglut3 expression level increased on day 7, thereby increasing the release of glutamate. The extent of GLAST increase may not be sufficient to fully take in glutamate due to increased Vglut3 expression level, resulting in excitotoxicity. Aminoglycosides, like noise, are excitotoxic to the peripheral auditory system (Kohrman et al., 2020). Kanamycin, an aminoglycoside, induced high expression of cochlear GLAST mRNA, which returned to normal until the 12th day after treatment (Matsuda et al., 1999). Although there is no significantly difference in expression of GLAST in our study, we found that there is the tendency for decreasing of excitotoxicity through increasing of GLAST. In our study, the expression pattern of GLAST induced by noise was consistent with that of kanamycin (Matsuda et al., 1999).

The interaction of Na^+^/K^+^-ATPase α1 with GLAST determines its important role in glutamate uptake. Ouabain, a selective inhibitor of Na^+^/K^+^-ATPase, can cause pathological changes in the rodent cochlea, mainly type I SGN damage (Lang et al., 2005; Fu et al., 2012; Yuan et al., 2014; Zhang et al., 2017; Schomann et al., 2018). Low spontaneous rate fiber damage in type I fibers is an important feature of synaptopathy (Furman et al., 2013; Kujawa and Liberman, 2015). Inhibition of Na^+^/K^+^-ATPase activity may significantly reduce the ability of GLAST to take-up glutamate (Rose et al., 2009). In our study, we found that the expression of GLAST and Na^+^/K^+^-ATPase α1 were almost identical within a week after noise exposure, which is reasonable for reducing excitotoxicity. The expression level of Na^+^/K^+^-ATPase α1 was reduced on day 30 after noise exposure, which may limit the function of GLAST, and the expression of Vglut3 increased, resulting in a pathological concentration of glutamate in the synaptic cleft, causing ribbon synaptopathy.

## Summary

In this study, we found an interaction between GLAST and Na^+^/K^+^-ATPase α1 in the cochlea of C57BL/6J mice. Based on the present results, we conclude that noise exposure influences ribbon synapses in two ways: (1) Noise briefly downregulated the expression of Vglut3 and upregulated the expression of GLAST and Na^+^/K^+^-ATPase α1, which trend to help to reduce glutamate toxicity in the synaptic cleft; (2) Noise damages ribbon synapses, and Vglut3 expression is upregulated for the normal transmission of auditory signals, but further downregulation of Na^+^/K^+^-ATPase α1 limits the uptake function of GLAST on day 30, which may further increase glutamate toxicity. Our study may provide a new approach for the prevention and treatment of ribbon synaptopathy.

## Data Availability Statement

The original contributions presented in the study are included in the article/supplementary material, further inquiries can be directed to the corresponding author/s.

## Ethics Statement

The animal study was reviewed and approved by Laboratory Animal Welfare Ethics Committee of Tianjin Institute of Environmental and Operational Medicine.

## Author Contributions

KM and AZ performed whole experimental work. KM wrote the manuscript. XS contributed to ABR experiment. XS, HY, KW, and YZ contributed to data analysis. HY, XG, and BC contributed to the experimental design. XG and BC revised the manuscript. KM and BC secured funding for the study. All authors discussed the results and approved the final manuscript.

## Conflict of Interest

The authors declare that the research was conducted in the absence of any commercial or financial relationships that could be construed as a potential conflict of interest.

## Publisher’s Note

All claims expressed in this article are solely those of the authors and do not necessarily represent those of their affiliated organizations, or those of the publisher, the editors and the reviewers. Any product that may be evaluated in this article, or claim that may be made by its manufacturer, is not guaranteed or endorsed by the publisher.
